# CARDIAC-FM: A Multimodal Foundation Model for Cardiovascular Risk Prediction Using ECG and Cardiac MRI

**DOI:** 10.64898/2026.03.16.26348526

**Published:** 2026-03-18

**Authors:** Fumin Li, Siting Li, Yuhan Qian, Bojun Chen, Jennifer A Brody, Vidhushei Yogeswaran, Kerri L Wiggins, Colleen M Sitlani, Joshua C Bis, Ali Shojaie, W T Longstreth, Bruce M Psaty, Geoffrey H Tison, Simon Du, James S Floyd, Ting Ye

**Affiliations:** 1. Department of Statistics, University of Washington, Seattle, WA, USA; 2. Department of Biostatistics, University of North Carolina at Chapel Hill, Chapel Hill, NC, USA; 3. Paul G. Allen School of Computer Science & Engineering, University of Washington, Seattle, WA, USA; 4. Department of Biostatistics, University of Washington, Seattle, WA, USA; 5. Cardiovascular Health Research Unit, Department of Medicine, University of Washington, Seattle, WA, USA; 6. Department of Epidemiology, University of Washington, Seattle, Washington, USA; 7. Department of Neurology, University of Washington, Seattle, Washington, USA; 8. Division of Cardiology, University of California-San Francisco, San Francisco, California, USA

## Abstract

Atrial fibrillation and heart failure impose substantial health burdens worldwide, yet existing prediction models lack sufficient accuracy and generalizability. We developed CARDIAC-FM, a multimodal foundation model that learns joint representations of 12-lead electrocardiogram (ECG) and cardiac magnetic resonance imaging (MRI) through contrastive learning. We trained CARDIAC-FM on 57,609 paired ECG-cardiac MRI samples from UK Biobank and evaluated it in two external cohorts: the Cardiovascular Health Study (CHS) and the Multi-Ethnic Study of Atherosclerosis (MESA). CARDIAC-FM consistently outperformed unimodal models across all cohorts, and jointly incorporating ECG features with established clinical risk scores yielded additive gains in discrimination, indicating that ECG and traditional risk factors capture complementary dimensions of cardiovascular risk. The learned representations improved prediction across a range of cardiovascular outcomes with minimal task-specific fine-tuning, reflecting real-world settings where many diseases have limited positive samples and lack dedicated risk models. Although trained on paired ECG and MRI data, CARDIAC-FM generates predictions using ECG alone or ECG combined with established risk scores, enabling broad clinical deployment without MRI. These findings demonstrate the promise of multimodal pre-training for generalizable cardiovascular risk prediction.

## Introduction

Atrial fibrillation and heart failure cause substantial cardiovascular morbidity and mortality worldwide, and their prevalence is projected to rise substantially over the coming decades^1–3^. Early identification of individuals at elevated risk is essential for targeted prevention, clinical decision-making, risk stratification across both clinical practice and trial design. However, existing risk prediction models based on traditional cardiovascular risk factors show varying discrimination and limited generalizability across populations^4–6^.

Deep learning applied to the resting 12-lead electrocardiogram (ECG) has shown promise for cardiovascular risk assessment. Convolutional neural networks trained on ECGs can detect subclinical cardiovascular conditions – including structural heart disease^7^, left atrial abnormalities^8,9^, ventricular dysfunction^10^ – and predict incident atrial fibrillation^11–13^ and other cardiovascular outcomes^14–16^. More recently, ECG foundation models trained with self-supervised learning on unlabeled data have further improved representation learning from electrocardiographic signals^17–19^. In parallel, deep learning applied to cardiac magnetic resonance imaging (MRI) has enabled automated cardiovascular disease screening and diagnosis^20–23^.

Despite these advances, current artificial intelligence (AI) models share several limitations that constrain clinical translation. Most models rely on a single modality, even though complementary data sources capture distinct and synergistic aspects of cardiac pathophysiology. Models are often developed in highly selected clinical cohorts, which limits their utility for risk prediction and population-based screening in apparently healthy individuals. Most models are also task-specific, requiring separate training for each outcome of interest. Finally, few have demonstrated robust generalizability across populations with different demographic characteristics, risk profiles, and healthcare contexts.

Recent multimodal approaches have begun to address the first limitation by integrating ECG with additional data modalities^23–25^ or combining imaging with clinical text^26^. However, these efforts have focused largely on identification of prevalent conditions rather than prospective prediction of cardiovascular events. To date, no foundation model has combined ECG and cardiac MRI using contrastive learning to predict incident cardiovascular events with demonstrated transferability across multiple large, independent cohorts and comparative performance against traditional risk scores.

Here, we introduce CARDIAC-FM (Contrastive Alignment for Risk Detection In Cardiovascular Foundation Model), a multimodal foundation model that integrates large-scale 12-lead ECG and cardiac MRI data from the UK Biobank. CARDIAC-FM uses a two-stage training framework. In the first stage, paired ECG and MRI data from 57,609 samples are used to pretrain modality-specific encoders and align their representations in a shared latent space through contrastive learning, enabling the ECG encoder to capture structural and functional information measured by MRI. In the second stage, a lightweight prediction head is added and fine-tuned for downstream outcome prediction, allowing efficient adaptation to various clinical endpoints without requiring separate, resource-intensive model training for each target condition. This stands in contrast to conventional approaches, where a dedicated model must be trained from scratch for every new outcome, demanding substantial labeled data and computational resources each time.

At inference, CARDIAC-FM can generate predictions using ECG alone or ECG combined with MRI, and can be further augmented with established risk scores, accommodating heterogeneous data availability in real-world clinical settings. We evaluated CARDIAC-FM for prediction of incident atrial fibrillation and heart failure in the UK Biobank and in two independent, multi-site prospective cohort studies in the United States – the Cardiovascular Health Study (CHS)^27^ and the Multi-Ethnic Study of Atherosclerosis (MESA)^28^. We also evaluated predictions across a broad range of additional cardiovascular outcomes in CHS and MESA. Across all cohorts, CARDIAC-FM consistently outperformed unimodal AI models, demonstrating superior generalizability. Jointly incorporating ECG and established risk scores through CARDIAC-FM yielded additive improvements in discrimination, indicating that ECG signals and traditional risk factors capture complementary aspects of cardiovascular risk. We further evaluated the pre-trained representations on additional cardiovascular outcomes in CHS and MESA — including myocardial infarction, ischaemic stroke, cardioembolic stroke, cardiovascular death, and all-cause death — under few-shot learning, reflecting the reality that labeled samples are often scarce in external cohorts. Strong performance under this limited-data regime confirms that multimodal pre-training captures a comprehensive representation of cardiac health that transfers effectively to diverse clinical endpoints. To our knowledge, CARDIAC-FM is the first ECG-MRI multimodal foundation model for prospective cardiovascular risk prediction with external validation across multiple independent cohorts. We publicly release the model code and pretrained weights to facilitate reproducibility and accelerate clinical translation of multimodal AI in cardiovascular medicine.

## Results

### UK Biobank Data and Participant Characteristics

At the time of analysis, resting 12-lead ECG and cardiac MRI data were available from 71,144 UK Biobank participants who had attended the baseline imaging visit. Participants had a mean age of 65.6 years (s.d. 7.8); 51.4% were female, and a total of 6,182 (8.7%) returned for repeat imaging, with a median inter-scan interval of 3.0 years (interquartile range 2.2–3.4 years), yielding 77,326 imaging visits (Supplementary Tables 1–2). ECG and MRI were acquired on the same day at each visit.

Among all the imaging visits, 70,424 visits had evaluable MRI and 67,661 had evaluable ECG data. Most visits (80.3%) had both modalities available, 7.1% had ECG only, and 10.7% had MRI only (Supplementary Table 1). After applying pre-specified quality control criteria (Methods), a total of 57,609 visits with paired, high-quality ECG and MRI data were retained for analysis. Participants with prevalent atrial fibrillation or heart failure at baseline were retained for pretraining but excluded from outcome prediction analyses. We randomly partitioned participants into training (60%), validation (5%), and test (35%) sets at the participant level, ensuring that all visits from the same individual were assigned to a single partition.

### Overview of Multimodal AI model

**Figure 1:**
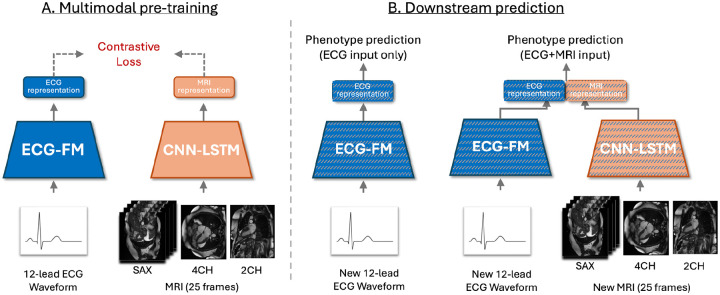
Overview of the CARDIAC-FM framework. **A**, Multimodal pretraining. ECG waveforms are processed through the ECG Foundation Model (ECG-FM) encoder and MRI (short-axis (SAX) stack, four-chamber (4CH), and two-chamber (2CH) views; 25 frames each) are processed through a convolutional neural network–long short-term memory (CNN-LSTM) encoder. The resulting representations are aligned in a shared latent space using contrastive loss. **B**, Downstream prediction. The pretrained ECG encoder can be used independently with ECG input alone (left) or combined with the pre-trained MRI encoder for multimodal prediction (right). Both configurations can further incorporate established clinical risk scores, enabling flexible deployment across clinical settings with varying data availability.

We developed a multimodal contrastive learning framework that integrates ECG and MRI data through self-supervised representation learning (Fig 1). During multimodal pretraining, modality-specific encoders extract features from raw ECG and MRI inputs, which are aligned in a shared latent space using a symmetric InfoNCE contrastive loss^29^. The model was trained on 34,596 samples with paired ECG and MRI data. For downstream outcome prediction, we excluded participants with prevalent disease at baseline, added a prediction layer to the learned representations, and fine-tuned the model for 20 epochs using supervised learning. The final model supports four input configurations: 1. ECG-only, 2.ECG + MRI, 3. ECG + Risk Score, 4. ECG + MRI + Risk Score, enabling deployment across clinical settings with varying imaging availability. Full methodological details are provided in Methods.

To evaluate the impact of modality combinations and clinical risk scores on predictive performance, we compared several model configurations. The comparator models were two established clinical risk scores derived from traditional risk factors (CHARGE-AF for atrial fibrillation and PREVENT-HF for heart failure)^6,30^, and an ECG-only foundation model without multimodal pretraining (ECG-FM). We then evaluated CARDIAC-FM under two input configurations: ECG only and combined ECG and MRI. For each AI model (ECG-FM and both CARDIAC-FM configurations), we further constructed variants that incorporated the corresponding clinical risk score (CHARGE-AF or PREVENT-HF) via late fusion. Performance was assessed on held-out test data using the area under the receiver operating characteristic curve (AUROC) and the area under the precision–recall curve (AUPRC).

### Fine-Tuning for Cardiac MRI Feature Prediction

We fine-tuned CARDIAC-FM with ECG-only input to predict MRI-derived cardiac structural and functional measures: left ventricular ejection fraction (LVEF), left ventricular mass (LVM), left ventricular end-diastolic volume (LVEDV), left ventricular end-systolic volume (LVESV), left atrial ejection fraction (LAEF), left atrial minimum volume (LAVmin), and left atrial maximum volume (LAVmax). ECG-predicted values were strongly correlated with direct imaging measures across all features (Pearson r=0.51–0.79; *R*^2^= 0.25–0.62; Supplementary Fig. 6; Supplementary Table 17). Correlations were generally higher for structural measures (LVM, *r* = 0.79; LVEDV, *r* = 0.72; LVESV, *r* = 0.71; LAVmin, *r* = 0.67) than for functional indices (LVEF, *r* = 0.51; LAEF, *r* = 0.55), with LAVmax showing intermediate performance (*r* = 0.57). These findings demonstrate that ECG representations learned through multimodal pretraining capture structural and functional cardiac information acquired with MRI, enabling imputation of imaging-derived features when cardiac MRI is unavailable.

### Fine-Tuning for Downstream Clinical Outcome Prediction

**Figure 2:**
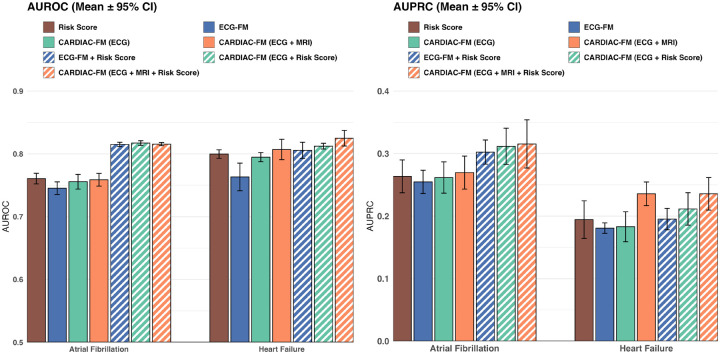
Prediction of incident atrial fibrillation and heart failure in the UK Biobank. Discrimination (AUROC, left) and precision–recall (AUPRC, right) for 5-year prediction of incident atrial fibrillation and heart failure in the held-out test set. Models compared include clinical risk scores alone (CHARGE-AF for atrial fibrillation; PREVENT-HF for heart failure), an ECG foundation model without multimodal pretraining (ECG-FM), and CARDIAC-FM under four input configurations: ECG only, ECG + MRI, ECG + clinical risk score, and ECG + MRI + clinical risk score. Error bars indicate 95% confidence intervals. CARDIAC-FM combined with clinical risk scores outperformed unimodal baselines across most comparisons.

We evaluated CARDIAC-FM for prediction of incident atrial fibrillation and heart failure at 5 years, comparing ECG-only and combined ECG–MRI inputs. All models were evaluated in a held-out UK Biobank test set restricted to participants with both modalities available, enabling fair comparison across input configurations (outcome rates in Supplementary Table 2; results in Fig. 2 and Supplementary Table 5).

For ECG-only models, ECG-FM (without multimodal pretraining) achieved AUROCs of 0.75 for atrial fibrillation and 0.76 for heart failure. CARDIAC-FM with ECG-only input showed comparable performance for atrial fibrillation but improved discrimination for heart failure (AUROC 0.80). Incorporating clinical risk scores into CARDIAC-FM substantially improved atrial fibrillation prediction but conferred smaller additional benefit for heart failure. Among models without MRI, CARDIAC-FM combining ECG with clinical risk score achieved the highest discrimination: AUROC 0.82 for atrial fibrillation and 0.81 for heart failure. These results indicate that ECG waveforms provide prognostic information independent of and complementary to traditional cardiovascular risk factors.

Adding MRI to CARDIAC-FM yielded minimal additional gains for atrial fibrillation prediction (AUROC and AUPRC differences <1 point) but improved heart failure prediction (AUROC gains of 1 point; AUPRC gains of 3–5 points). The full CARDIAC-FM model integrating ECG, MRI, and clinical risk scores achieved AUROCs of 0.82 for atrial fibrillation and 0.83 for heart failure.

To assess whether CARDIAC-FM generalized across clinically relevant subgroups, we evaluated model performance stratified by age (<65 versus ≥65 years) and sex (Supplementary Fig. 3; Supplementary Table 10). CARDIAC-FM demonstrated consistent discrimination across all subgroups for both atrial fibrillation and heart failure, with AUROC differences typically <5 percentage points and no substantial effect modification by age or sex.

### External Validation in CHS and MESA Cohorts

**Figure 3:**
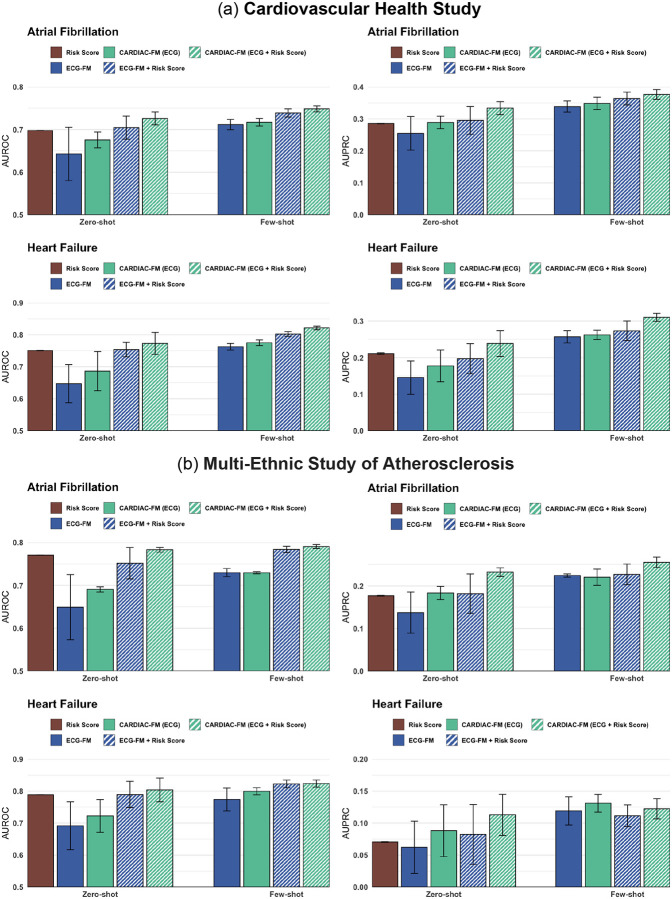
External validation of CARDIAC-FM in the (a) Cardiovascular Health Study and (b) Multi-Ethnic Study of Atherosclerosis. Zero-shot and few-shot prediction performance for 5-year incident atrial fibrillation and heart failure, evaluated using AUROC and AUPRC. Models were pretrained on UK Biobank and externally validated on CHS and MESA. Zero-shot denotes direct application without cohort-specific training; few-shot denotes fine-tuning on 20% of the external cohort data, respectively. Error bars represent 95% confidence intervals. CARDIAC-FM (ECG + Risk Score) achieved the strongest performance across all settings.

We evaluated CARDIAC-FM in two independent US prospective cohort studies: the Cardiovascular Health Study (CHS) and the Multi-Ethnic Study of Atherosclerosis (MESA). CHS enrolled 5,888 adults aged 65 years or older in 1989–1990 and 1992–1993, including predominantly White participants and a smaller proportion of African American individuals, with a mean age of 73 years at baseline. MESA enrolled 6,814 adults aged 45–84 years from four racial and ethnic groups (White, African American, Hispanic, and Chinese American) in 2000–2002, all free of clinical cardiovascular disease at enrollment, with a mean age of 62 years. Both cohorts differed from UK Biobank imaging participants (mean age 65 years) in age distribution and racial and ethnic composition but provided longitudinal surveillance for atrial fibrillation events and central physician adjudication of heart failure, enabling robust assessment of generalizability. In CHS, participants underwent standardized resting 12-lead ECGs at the baseline examination, whereas in MESA participants underwent both ECG and MRI at baseline. We therefore evaluated CARDIAC-FM (ECG only) and CARDIAC-FM (ECG + Risk Score) in both external cohorts (Fig. 3; Supplementary Tables 6–7).

We first evaluated model generalizability in CHS, which included 10,058 ECG recordings from 5,806 unique participants at baseline and year-7 visits (outcome rates in Supplementary Table 3). In zero-shot evaluation, models trained on UK Biobank were applied directly without fine-tuning, reflecting the most common practical scenario of applying pretrained models to new populations without additional training. Traditional risk scores were evaluated in the zero-shot setting only, as they are established models with fixed coefficients. CARDIAC-FM (ECG + Risk Score) yielded the strongest performance across both outcomes (AUROC 0.73 and AUPRC 0.33 for atrial fibrillation; AUROC 0.77 and AUPRC 0.24 for heart failure). Compared with the clinical risk scores alone, this represented a 3-point increase in AUROC and 5-point increase in AUPRC for atrial fibrillation, and a 2-point increase in AUROC and 3-point increases in AUPRC for heart failure. We next evaluated few-shot fine-tuning using 20% of CHS data. This minimal adaptation improved both AUROC and AUPRC across all configurations, with CARDIAC-FM (ECG + Risk Score) achieving the strongest performance under all conditions (AUROC 0.75 and AUPRC 0.38 for atrial fibrillation; AUROC 0.82 and AUPRC 0.31 for heart failure; Fig. 3a). This level of performance is comparable to that observed in the held-out UK Biobank test set, demonstrating that the foundation model can be efficiently adapted to new populations. Across all experiments, CARDIAC-FM consistently outperformed ECG-FM given the same inputs, demonstrating the benefit of multimodal pretraining in improving generalization.

**Figure 4:**
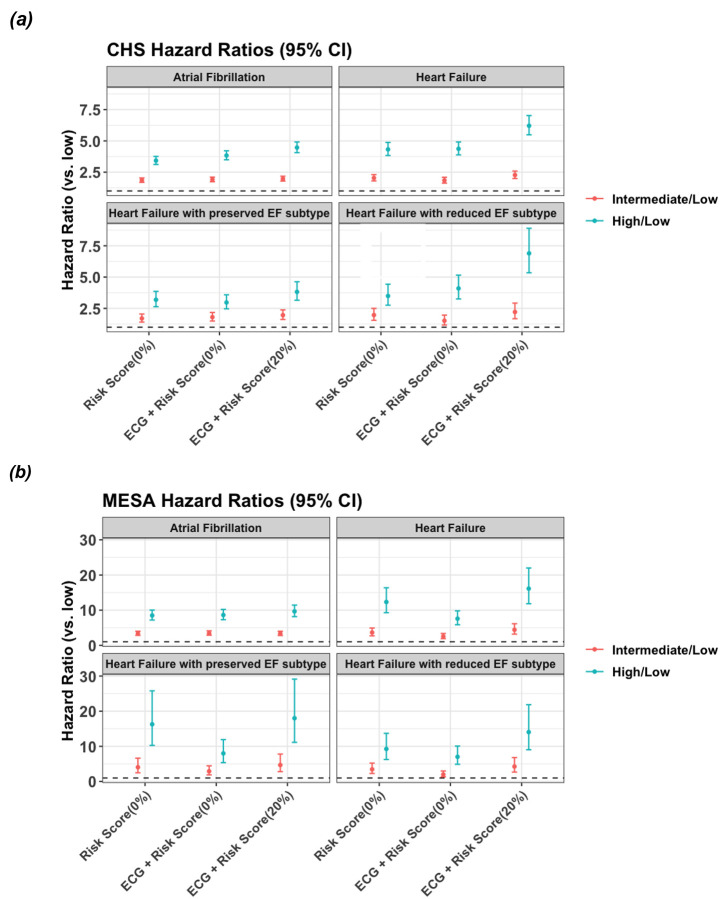
Risk stratification performance of CARDIAC-FM in external validation cohorts. Hazard ratios for 5-year incident atrial fibrillation, heart failure, heart failure with preserved ejection fraction (HFpEF), and heart failure with reduced ejection fraction (HFrEF) in the Cardiovascular Health Study (CHS; **a**) and 5-year incident atrial fibrillation and heart failure in the Multi-Ethnic Study of Atherosclerosis (MESA; **b**). Participants were stratified into risk tertiles based on clinical risk scores alone (CHARGE-AF for atrial fibrillation; PREVENT-HF for heart failure) or CARDIAC-FM (ECG + Risk Score) under zero-shot and 20% fine-tuning conditions. Hazard ratios were estimated using univariable Cox proportional hazards models with the low-risk tertile as reference. Teal points indicate high-risk versus low-risk tertile; red points indicate intermediate-risk versus low-risk tertile. Error bars indicate 95% confidence intervals. In CHS, CARDIAC-FM outperformed clinical risk scores in zero-shot evaluation, with further improvement upon fine-tuning. In MESA, lower event rates, particularly for heart failure, resulted in wider confidence intervals; CARDIAC-FM achieved similar performance to clinical risk scores for atrial fibrillation but underperformed for heart failure in zero-shot evaluation, with comparable or superior risk stratification after fine-tuning.

Results in MESA were broadly consistent, with CARDIAC-FM (ECG + Risk Score) achieving the highest discrimination across all settings. In particular, CARDIAC-FM yielded absolute improvements in AUPRC of 4–5% for both outcomes under zero-shot evaluation and 5–8% with few-shot fine-tuning (Fig. 4b). Subgroup analyses stratified by age and sex demonstrated stable performance across both cohorts (Supplementary Figs. 4 and 5; Supplementary Tables 11 and 14).

We also evaluated CARDIAC-FM’s risk stratification performance in CHS and MESA (Fig. 4; Supplementary Tables 12 and 15). Using predicted risk from the ECG + Risk Score model, we stratified the test set into tertiles and estimated hazard ratios via univariable Cox regression. In CHS, zero-shot CARDIAC-FM outperformed clinical risk scores alone, with high-risk tertiles showing hazard ratios of 3.83 (95% CI:3.50–4.21) for atrial fibrillation and 4.37 (95% CI: 3.88–4.91) for heart failure versus the low-risk reference. Few-shot fine-tuning further improved discrimination: with 20% fine-tuning, hazard ratios increased to 4.47 (95% CI: 4.06–4.91) for atrial fibrillation and 6.21 (95% CI: 5.49–7.03) for heart failure. Because CHS had sufficient events for heart failure subtype analysis, we examined differential benefits of ECG integration. HFrEF showed the largest improvement, consistent with prominent electrical abnormalities from structural remodeling, whereas HFpEF demonstrated more modest gains. In MESA, lower event rates, particularly for heart failure (Supplementary Table 4), yielded wider confidence intervals. Zero-shot CARDIAC-FM achieved risk stratification similar to clinical scores for atrial fibrillation but underperformed for heart failure; fine-tuning restored comparable or superior performance across all outcomes.

To assess whether ECG-predicted MRI parameters capture prognostically relevant information, we examined associations between zero-shot CARDIAC-FM-predicted MRI parameters and incident cardiovascular events in CHS, adjusting for traditional risk factors (Supplementary Fig. 1; Supplementary Table 13). Predicted left atrial volumes (LAVmax, LAVmin) and left ventricular structural measures (LVEDV, LVESV, LVM) were positively associated with incident atrial fibrillation and heart failure, whereas predicted ejection fractions (LAEF, LVEF) showed protective associations. These patterns were more pronounced for HFrEF, with predicted ventricular volumes and mass showing hazard ratios of 1.43–1.64 and LVEF demonstrating the strongest protective association (HR ~0.67). In contrast, HFpEF showed minimal associations across all predicted parameters. These analyses were possible because CARDIAC-FM enabled inference of MRI-derived features from ECG alone, allowing examination of cardiac structure–disease associations in a cohort without cardiac imaging. In MESA, where cardiac MRI was performed, associations between predicted and measured MRI parameters with incident cardiovascular events were largely consistent (Supplementary Fig. 2; Supplementary Table 16), validating the prognostic relevance of ECG-derived structural estimates.

### Evaluation of multimodal pre-trained representations across a range of cardiovascular outcomes

We hypothesized that multimodal contrastive pre-training would yield a representation of cardiac health that can improve prediction of a broad range of cardiovascular outcomes. To test this, we leveraged the rigorously adjudicated outcomes available in CHS and MESA and evaluated CARDIAC-FM (ECG-only) on five additional endpoints: myocardial infarction, ischaemic stroke, cardioembolic stroke (the ischemic stroke subtype most closely linked with atrial fibration), cardiovascular mortality, and all-cause mortality. The event rates for these outcomes are reported in Supplementary Tables 3–4. For each outcome, we assessed prediction at both 5- and 10-year horizons, except for cardioembolic stroke where only 10-year prediction was evaluated due to insufficient events at shorter follow-up. We fine-tuned the pre-trained CARDIAC-FM and ECG-FM encoders using ECG-only input and only 20% of data from each cohort, reflecting a data-scarce setting typical of deploying foundation models to new clinical populations with limited outcome annotations.

**Figure 5:**
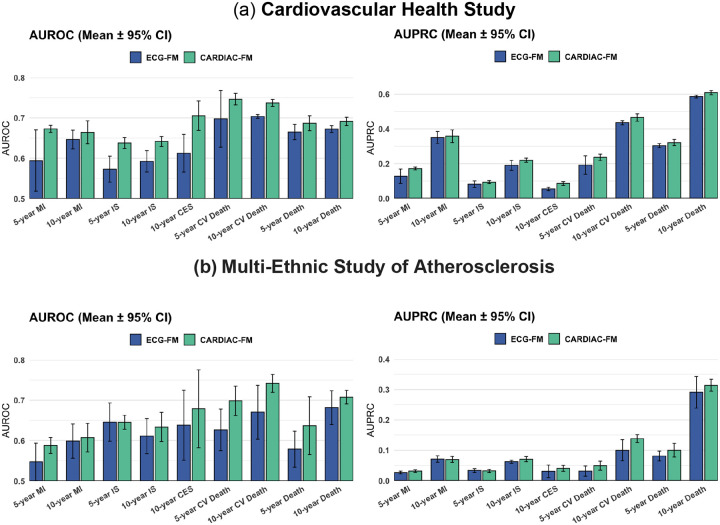
Evaluation of pre-trained representations across a broad range of cardiovascular outcomes. AUROC (left) and AUPRC (right) for CARDIAC-FM (blue) and ECG-FM (red) across five cardiovascular outcomes at 5- and 10-year prediction horizons in CHS (**a**) and MESA (**b**). Outcomes include myocardial infarction (MI), ischaemic stroke (IS), cardioembolic stroke (CES; 10-year only due to limited events at shorter follow-up), cardiovascular death (CV death), and all-cause death (death). Both models used ECG-only input and were fine-tuned using 20% of data from each cohort. CARDIAC-FM consistently outperformed ECG-FM across all outcomes and time horizons in both cohorts. Error bars indicate 95% confidence intervals.

Under minimal fine-tuning, CARDIAC-FM consistently outperformed ECG-FM in both AUROC and AUPRC across all outcomes and time horizons in both cohorts, with the exception of 5-year incident IS in MESA, where performance was comparable between the two models (Fig. 5, Supplementary Tables 8–9), with AUROC improvement ranging from 0.02 to 0.09 in CHS, and 0.00 to 0.07 in MESA. These findings indicate that multimodal pre-training with cardiac MRI encodes a broad representation of cardiac structure and function into the ECG encoder that transfers to diverse clinical endpoints with minimal task-specific supervision.

## Discussion

In this study, we present CARDIAC-FM, the first large-scale multimodal foundation model integrating ECG waveform data and cardiac MRI for prospective cardiovascular event prediction. A key finding is that multimodal pre-training enhances model performance even when only ECG is available at inference, demonstrating that cross-modal representation learning enriches unimodal ECG features without requiring MRI during deployment. These improvements likely reflect an enhanced capacity of the ECG encoder to capture features predictive of cardiac structure and function, information learned through alignment with MRI during pre-training that is particularly relevant to heart failure pathophysiology, without prespecifying any imaging parameters. Importantly, the benefits of multimodal pre-training extended beyond the primary endpoints of atrial fibrillation and heart failure: when evaluated on a broad range of additional cardiovascular outcomes in CHS and MESA, including myocardial infarction, ischaemic stroke, cardioembolic stroke, cardiovascular death, and all-cause death, CARDIAC-FM consistently outperformed its unimodal counterpart with minimal fine-tuning, indicating that the learned representations capture a comprehensive picture of cardiac health rather than features specific to any single disease process.

In our analyses, we compared CARDIAC-FM with established clinical risk scores that are widely used in research and, to a lesser degree, in clinical practice. There are distinct advantages to estimating cardiovascular risk from a single, widely available biological measurement such as the resting 12-lead ECG. Clinical risk scores incorporate readily available variables including age, sex, and body mass index, yet other components such as diabetes assessment or documentation of clinical cardiovascular disease involve costly laboratory or diagnostic testing that may be difficult for many patients to access. Risk prediction based on a single biological measurement that synthesizes key features of cardiac structure has the potential to mitigate bias arising from incomplete ascertainment of predictors, which often relates to socioeconomic position and access to care. More importantly, beyond a small number of common conditions, most cardiovascular diseases do not have dedicated risk prediction models. By contrast, our model can use ECG as the sole input to estimate risk for a wide range of cardiovascular outcomes. Even when only a limited number of positive samples are available, minimal task-specific fine-tuning enables the model to adapt to new disease endpoints, bypassing the need to develop separate disease-specific risk models from scratch.

Beyond prediction, CARDIAC-FM functions as a cross-modality translator, inferring MRI-derived structural markers from ECG data. Cardiac MRI is expensive, requires specialized infrastructure, and was not performed in most epidemiological cohorts. By generating MRI-derived structural estimates from routine ECG recordings, CARDIAC-FM enables population-scale investigation of cardiac structure and its relationship to clinical outcomes in cohorts that lack imaging data. We demonstrated this capability in CHS, where cardiac MRI was not acquired, and validated the prognostic relevance of the predicted structural parameters against measured MRI values in MESA.

CARDIAC-FM supports two inference modes: an ECG-only mode that is broadly deployable in routine clinical settings and a comprehensive ECG+MRI mode that maximizes predictive performance when imaging is available. Both modes can incorporate established clinical risk scores via late fusion. Across three independent cohorts, CARDIAC-FM generally outperformed both unimodal deep learning models and clinical risk scores, highlighting the robustness and clinical relevance of multimodal training. The model demonstrated strong generalizability across diverse populations, and its efficient fine-tuning paradigm enabled rapid adaptation with limited labeled data.

Our results also clarify the distinct and complementary roles of ECG and MRI in predicting different cardiovascular conditions. Raw ECG waveforms exhibit independent predictive value that extends beyond traditional ECG markers; for example, PR interval is not a strong predictor of atrial fibrillation, yet raw ECG waveforms contain richer temporal and morphological information that contributes to prediction. We further find that adding MRI on top of raw ECG contributes little additional value for atrial fibrillation prediction. A likely explanation is that atrial fibrillation is an electrical disorder, and ECG waveforms can capture early electrical instability and subclinical atrial fibrillation episodes that precede or occur without structural abnormalities on MRI. In contrast, for heart failure, multimodal learning provides clear benefits. Heart failure encompasses several phenotypes and often involves subtle, subclinical structural changes, such as early remodeling, fibrosis, or impaired contractility that are challenging to detect from ECG alone. MRI is highly sensitive to these structural features, and training with combined ECG and MRI data enhances heart failure prediction even when only ECG is available at inference. Although our current MRI model provides informative features, future improvements in imaging encoders may further strengthen heart failure prediction.

These findings suggest that multimodal foundation modelling is a promising approach to cardiovascular risk assessment. By supporting flexible inference modes, leveraging complementary information across modalities, and maintaining strong performance with minimal labeled data across a range of cardiovascular endpoints, CARDIAC-FM provides a scalable framework for integrating heterogeneous clinical data into predictive cardiovascular medicine.

## Online Methods

### UK Biobank

The UK Biobank (UKB) is a cohort study that recruited 500,000 participants aged 40 to 69 years from the United Kingdom between 2006 and 2010^31^. The baseline assessment included comprehensive questionnaires, blood and urine collection for biochemical measurements, anthropometry, and interviews. All participants provided electronic informed consent at the UK Biobank assessment centers. The UKB received ethics approval from the North West Multi-Centre Research Ethics Committee.

Longitudinal follow-up for health outcomes of atrial fibrillation, and heart failure was performed through linkages with national databases on hospitalizations, primary care records, and death registries. Incident atrial fibrillation events were identified from self-reports, nurse interviews, hospital admission codes, death register cause-of-death codes, and UK Biobank first occurrence data. Heart failure was identified solely from diagnosis codes and cause-of-death codes (Supplementary Table 18).

We defined binary prediction outcomes at a 5-year time horizon following the imaging visit. For each participant, we coded atrial fibrillation status as positive if the participant had no prior diagnosis of atrial fibrillation at the imaging visit and developed incident atrial fibrillation within 5 years, as negative if the participant completed 5-year follow-up without an event, and as missing otherwise. We excluded participants with prevalent atrial fibrillation at the imaging visit from atrial fibrillation prediction analyses. We defined heart failure analogously and excluded participants with prevalent disease at the imaging visit from respective analyses.

Participants whose imaging visits occurred after the UKB data censoring dates were excluded. Follow-up for this study extended through 2022. Hospital data were available until October 2022 for England, August 2022 for Scotland, and May 2022 for Wales. Death record data was censored in November 2022 across all sites.

Traditional cardiovascular risk factors were measured at the imaging visit or, when unavailable, at the most recent prior visit. Covariates included age, sex, height, weight, body mass index (BMI), systolic and diastolic blood pressure, total cholesterol, high-density lipoprotein cholesterol, estimated glomerular filtration rate (eGFR; calculated using the 2009 Chronic Kidney Disease Epidemiology Collaboration creatinine equation), diabetes mellitus, current smoking status, prevalent myocardial infarction, and current use of antihypertensive and lipid-lowering medications. These variables comprise the CHARGE-AF and American Heart Association PREVENT-HF (Predicting Risk of Cardiovascular Disease EVENTs equation for heart failure).

### Cardiovascular Health Study

The Cardiovascular Health Study (CHS) is a prospective cohort study of risk factors for cardiovascular diseases in adults aged 65 years and older, recruited from four field centers in the United States (Sacramento, CA; Hagerstown, MD; Winston-Salem, NC; Pittsburgh, PA)^27^. Between June 1989 and June 1990, 5,201 participants were recruited from random samples of Medicare eligibility lists. An additional 687 Black participants were recruited between November 1992 and June 1993. Study participants were seen in the clinic annually between enrollment and 1998–99 and were contacted by telephone at 6-month intervals through June 2015 to collect information about health status, hospitalizations, and medication use^32^. The study was approved by the institutional review boards of all participating sites, and all study participants provided informed consent.

Atrial fibrillation events were identified using a combination of ECG findings from clinic visits through 1999, hospital discharge records indicating atrial fibrillation, and Medicare inpatient, outpatient, and physician (carrier) claims data. Resting 12-lead ECGs were performed at baseline and follow-up visits, with centralized readings to classify atrial fibrillation according to standardized criteria. For atrial fibrillation identified through Medicare data, diagnosis required either ECG evidence, one inpatient claim, or two outpatient or physician claims within 365 days using International Classification of Diseases, Ninth Revision (ICD-9) codes 427.31 or 427.32 in any diagnostic position.

Assessment of heart failure events in CHS has been previously described^33^. All potential heart failure events were identified through in-person visits or telephone interviews. These events were then adjudicated by the central events committee. Adjudication of heart failure was based on a physician diagnosis of heart failure, documented symptoms and signs of heart failure, use of medical treatment for heart failure, and supportive findings on diagnostic testing.

Methods for clinical ischemic stroke adjudication in CHS has been previously described^34^. In summary, an expert events committee, including neurologists from the study sites and CHS coordinating center, along with a neuroradiologist from the imaging reading center, reviewed the cases. Ischemic stroke was characterized by the sudden onset of a focal neurological deficit persisting for at least 24 hours, supported by compatible imaging findings on computed tomography scan or cardiac magnetic resonance imaging, and without evidence of intracranial hemorrhage or a nonvascular underlying cause. The adjudication committee categorized ischemic strokes into four subtypes based on the presumed infarction mechanism: cardioembolic, small vessel, large-artery atherosclerotic, or unknown/other causes.

Myocardial infarction (MI) was adjudicated to have occurred in the presence of evolving Q-wave MI or cardiac pain plus abnormal enzymes and either an evolving ST-T pattern or new left bundle branch block.

Details of surveillance methods and disease classification in CHS have been described previously^35^. Since participants were enrolled in the study, deaths were identified through phone calls, annual clinic visits, or local obituaries. Cause of death was adjudicated by a centralized adjudication committee, which reviewed information from obituaries, death certificates, medical records, and proxy interviews^36^. Cardiovascular death is defined as death from coronary artery disease.

### Multi-Ethnic Study of Atherosclerosis

The Multi-Ethnic Study of Atherosclerosis (MESA) is a prospective, population-based observational cohort study of 6,814 men and women representing four racial/ethnic groups (white, African American, Hispanic, and Chinese-American), aged 45–84 years and free of clinical cardiovascular disease at enrollment^28^. Study participants were recruited in 2000–2 at 6 field centers in the US (Baltimore, MD; Chicago, IL; Forsyth County, NC; Los Angeles, CA; New York, NY; and St. Paul, MN). At telephone contacts every 9–12 months during follow-up, participants were asked to identify new hospitalizations and diagnoses, and medical records were obtained. Institutional review boards of all field centers approved the study protocol, and all participants gave written informed consent.

Clinically recognized atrial fibrillation was identified by an ICD hospital discharge diagnosis code (version 9: 427.31 or 427.32; version 10: I48) in any position; and for those enrolled in fee-for-service Medicare, by an inpatient, outpatient, or physician claim with an atrial fibrillation code^37^. A panel reviewed medical records and adjudicated heart failure events according to standardized criteria. The present study included probable or definite heart failure events. Probable heart failure required heart failure symptoms, a physician diagnosis of heart failure, and treatment. Definite heart failure required at least one objective feature of heart failure, including abnormalities on chest X-ray, echocardiography, or ventriculography.

Methods for clinical ischemic stroke adjudication in MESA has been previously described^38^. In brief, information about all new cardiovascular conditions, hospital admissions, cardiovascular outpatient diagnoses, treatments, and deaths were obtained. Two vascular neurologists from the MESA study events committee independently reviewed all medical records for end point classification and assignment of incidence dates. They reviewed and classified stroke as present if there was a focal neurologic deficit lasting 24 hours or until death or, if <24h, there was a clinically relevant lesion on brain imaging and there was no nonvascular cause. Patients with focal neurological deficits secondary to brain trauma, tumor, infection, or other nonvascular cause were excluded. Ischemic strokes were distinguished from hemorrhagic stroke using findings on imaging, surgery, autopsy, or some combination of these. Ischemic stroke subtypes were assigned based on an extension of the Trial of Org 10172 in Acute Stroke Treatment (TOAST) criteria to try to reduce the number classified as undetermined.

An myocardial infarction in MESA required either abnormal cardiac biomarkers (2 times upper limits of normal) regardless of pain or ECG findings; evolving Q waves regardless of pain or biomarker findings; or a combination of chest pain, ST-T evolution or new left bundle branch block, and biomarker levels 1 to 2 times upper limits of normal^39^.

Hard cardiovascular disease events in MESA were required to be symptomatic and included myocardial infarction (MI), resuscitated cardiac arrest, stroke, and cardiovascular death. Incident heart failure events were recorded. All deaths were identified. For potential cardiovascular deaths, cause was assigned through committee review. For other deaths, the underlying cause was obtained through state or city vital statistics departments^40^.

### Cardiac magnetic resonance imaging

The UK Biobank Imaging Study, initiated in 2014, includes magnetic resonance imaging of the brain, abdomen, and heart^41^. The first 5,065 cardiac MRI scans were manually analyzed, and LA contours at end-systole and end-diastole were traced^42^. Bai et al^43^. used these annotated images to develop a fully automated convolutional network pipeline for large-scale quantification of cardiac structure and function. The algorithm’s accuracy was evaluated using technical metrics (Dice coefficient, contour distance, and Hausdorff distance) and validated by clinical experts. This publicly available algorithm was applied to derive MRI parameters, including left ventricular ejection fraction (LVEF), end-diastolic and end-systolic volumes (LVEDV, LVESV), left ventricular mass (LVM), left atrial emptying fraction (LAEF), and minimum and maximum left atrial volumes (LAVmin, LAVmax).

We preprocessed multi-view cine MRI scans (two-chamber [2CH], four-chamber [4CH], and short-axis [SAX]) before input to our multimodal AI pipeline. The 2CH and 4CH views each comprised a single slice, whereas the SAX view usually comprised 11–12 slices. Each MRI slice contained 50 time points; we subsampled every other time point to reduce computational requirements, yielding 25 time points per slice. Using segmentation masks to identify the left ventricular center, we extracted a 128×128-pixel patch from each image, removing background information while preserving the cardiac region.

We then applied standardized preprocessing to all slices to match the input format of a pretrained CNN-LSTM model^20^: (1) zero-padding to 210×210 pixels to ensure uniform dimensions; (2) normalization using the mean and standard deviation computed across all views within each sample; and (3) resizing to 64×64 pixels using bilinear interpolation. Our model required six slices per sample; therefore, we retained only samples containing 2CH, 4CH, and at least four SAX slices (after sampling every other slice from all SAX views) with valid anatomical segmentation masks, yielding 65,480 samples with evaluable MRI data.

### Electrocardiograms

In UK Biobank, each ECG trace comprises 5,000 uniformly sampled data points per lead over 10 seconds at 500 Hz. We parsed 12-lead ECG signals, including all standard leads (I, II, III, aVR, aVL, aVF, V1–V6).

We excluded ECGs with poor quality, suspected lead reversal, undetermined rhythm, or implausible ventricular rates (<20 or >140 bpm). We also excluded ECGs showing atrial fibrillation, atrial flutter, or pacemaker activity. After quality control, 67,661 samples with valid 12-lead ECG data were retained for analysis.

To remove baseline wander, we applied locally weighted scatterplot smoothing (LOWESS) independently to each lead. For each 5,000-point trace, we fitted a LOWESS curve using the signal as the response variable and sample index as the predictor with a smoothing fraction of 0.1. The resulting smoothed curve, representing low-frequency drift, was subtracted from the raw signal to obtain baseline-corrected ECG data.

In CHS and MESA, resting 10-second 12-lead ECGs were recorded at 500 Hz using MAC PC ECG machines (Marquette Electronics, Milwaukee, WI) at baseline and selected follow-up visits. All ECGs were centrally reviewed for technical errors and quality at the Epidemiological Cardiology Research Center (EPICARE), Wake Forest University School of Medicine (Winston-Salem, NC), using General Electric 12-SL software (GE Healthcare, Milwaukee, WI) on MUSE and Magellan workstations. In the ECG signals from both the CHS and MESA cohorts, no significant baseline wander was observed. ECG signals from both cohorts showed no significant baseline wander. CHS ECG amplitudes were rescaled to align with UK Biobank reference values, whereas MESA ECG amplitudes were already comparable and required no adjustment.

### Multimodal AI development

#### CARDIAC-FM framework.

The CARDIAC-FM framework comprised a two-stage pipeline. In the first stage, modality-specific encoders extracted features from ECG and MRI inputs, which were aligned in a shared latent space using symmetric InfoNCE contrastive loss^29^. This objective encouraged both encoders to learn shared, cross-modal representations. We used state-of-the-art open-source models as encoders: ECG Foundation Model (ECG-FM) for ECG data and a convolutional neural network–long short-term memory (CNN-LSTM) architecture for MRI data. The ECG-FM encoder was initialized with pretrained weights and used the global representation after average pooling. The MRI encoder was initialized with weights pretrained for binary classification of left ventricular ejection fraction (LVEF ≤50% versus >50%) and used the final LSTM layer output as the representation. In the second stage, a prediction layer was added to the learned representations and the entire model was fine-tuned with supervised learning for clinical outcome prediction. The final model accepts two input configurations—ECG-only or combined ECG+MRI—enabling broad clinical applicability across settings with varying data availability.

#### Loss objectives for multimodal pretraining.

The learned representations were trained using symmetric InfoNCE loss across modalities^29^. Consider a batch containing *B* paired samples, where each sample consists of one ECG input and one MRI input, denoted by ${\left\{\left(x_{i}^{ECG},x_{i}^{MRI}\right)\right\}}_{i=1}^{B}$. The modality-specific encoders produce feature representations:

$$u_{i}=f_{MRI}\left(x_{i}^{MRI}\right)\in R_{d},\;v_{i}=f_{ECG}\left(x_{i}^{ECG}\right)\in R_{d}.$$

The similarity function between representations is defined as:

$$s_{ij}=\frac{<u_{i},v_{j}>}{\tau }$$

Where τ denotes the temperature parameter. The InfoNCE Loss for contrastive learning is then computed as:

$$L_{MRI\to ECG}=-\frac{1}{B}\overset{B}{\underset{i=1}{\sum }}\text{log}\frac{\text{exp}\left(s_{ii}\right)}{\overset{B}{\underset{j=1}{\sum }}\text{exp}\left(s_{ij}\right)}$$

$$L_{ECG\to MRI}=-\frac{1}{B}\overset{B}{\underset{i=1}{\sum }}\text{log}\frac{\text{exp}\left(s_{ii}\right)}{\overset{B}{\underset{j=1}{\sum }}\text{exp}\left(s_{ji}\right)}$$

$$L_{\text{InfoNCE}}=\frac{1}{2}\left(L_{MRI\to ECG}+L_{ECG\to MRI}\right)$$

This loss is computed across ECG-MRI pairs within each batch, pulling together embeddings from paired modalities of the same subject while pushing apart embeddings from different individuals. This contrastive learning step established the foundation for subsequent fine-tuning on downstream prediction tasks.

#### Model architecture.

The ECG-FM encoder comprised a multi-layer convolutional neural network (CNN) feature extractor and a BERT-Large transformer encoder (https://github.com/bowang-lab/ECG-FM). The CNN-LSTM encoder consisted of two DenseNet-40–12 CNNs and two LSTM encoders (https://github.com/MedAI-Vision/CMR-AI). During multimodal pretraining, only the transformer component of ECG-FM and the LSTM component of CNN-LSTM were trainable. A linear projection layer was appended to each encoder to align feature dimensions to 512; for ECG-FM, this layer reduced dimensionality from 768 to 512.

#### Training Details.

Multimodal pretraining was performed on four RTX 2070 8GB GPUs with a batch size of 32 for 20 epochs using the AdamW optimizer (β_1_ = 0.9, β_2_ = 0.98, ε = 1×10^−6^, weight decay = 0.05). Learning rate followed a cosine annealing schedule with base rate 5×10^−6^ and warm-up length of 50 steps. The temperature parameter in the InfoNCE loss was trainable and initialized at 0.07.

For downstream supervised fine-tuning, all ECG encoder parameters were trainable, along with the LSTM component and projection layer of the MRI encoder. Downstream fine-tuning on UK Biobank data was performed on a single RTX 2070 8GB GPU with a batch size of 4 for 20 epochs using the AdamW optimizer (β_1_ = 0.9, β_2_ = 0.98, ε = 1×10^−6^, weight decay = 0.01) with a linear warm-up schedule (base learning rate 5×10^−6^, warm-up length 50 steps). Model checkpoints were selected based on the highest AUROC on the validation set. No hyperparameter tuning was performed beyond optimizer and learning rate scheduler selection.

For the risk factors in the clinical risk models, all predictors except sex contained varying degrees of missingness (approximately 1–18%). We applied multiple imputation using chained equations (MICE) before model fitting, generating four imputed datasets. When combining risk factors with the ECG or ECG+MRI model, we employed a late-fusion strategy in which established risk scores and the ECG-based or ECG+MRI-based model were integrated using a final logistic regression classifier.

For all models, we repeated training using four different random seeds, yielding four independent sets of predicted values. For the established risk scores, we derived the four sets of predictions from the four independently completed MICE-imputed datasets. We calculated confidence intervals for AUC estimates from the standard deviation across the four realizations, using the mean ± 1.96 × SD to approximate 95% intervals. These confidence intervals quantify variability due to random seed, conditional on the dataset.

#### Subgroup analysis.

We performed subgroup analyses stratified by sex (male versus female) and age (<65 versus ≥65 years). Model performance was evaluated separately within each subgroup across the UK Biobank, Cardiovascular Health Study, and Multi-Ethnic Study of Atherosclerosis cohorts.

### Statistical Analysis

#### Cox proportional hazards analysis across risk strata.

We performed Cox regression analyses to evaluate the prognostic value of three model configurations: risk scores alone, CARDIAC-FM (ECG + risk scores), and CARDIAC-FM (ECG + MRI + risk scores). For each model and outcome (atrial fibrillation and heart failure), predicted risk scores (averaged across four random seeds) were stratified into tertiles representing high-, intermediate-, and low-risk groups, with the low-risk tertile serving as the reference. We estimated hazard ratios and 95% confidence intervals for the high- and intermediate-risk groups using univariable Cox proportional hazards models.

#### Cox proportional hazards analysis across MRI features.

We performed Cox regression analyses to evaluate the prognostic value of cardiac MRI features, including left atrial volumes (LAVmax, LAVmin), left atrial emptying fraction (LAEF), left ventricular volumes (LVEDV, LVESV), left ventricular ejection fraction (LVEF), and left ventricular mass (LVM). We estimated hazard ratios using Cox proportional hazards models adjusted for traditional risk factors. Because these models included multiply imputed risk factors, we calculated confidence intervals using Rubin’s rules.

In UK Biobank, Cox models for atrial fibrillation and heart failure were constructed separately using two distinct sets of predictors: measured MRI features and CARDIAC-FM-predicted MRI features derived from ECG. Hazard ratios were estimated from both model sets to compare prognostic performance.

In the Cardiovascular Health Study and Multi-Ethnic Study of Atherosclerosis, Cox models were fitted for atrial fibrillation, heart failure and their respective subtypes using CARDIAC-FM-predicted MRI features derived from models trained on UK Biobank. In MESA, where cardiac MRI was performed, models using measured MRI features were also constructed, enabling direct comparison between predicted and measured structural parameters.

## Supplementary Material

Supplement 1

Supplement 2

## Data Availability

UK Biobank data are made available to researchers from research institutions as described at this site: https://www.ukbiobank.ac.uk/enable-your-research. Data for Cardiovascular Health Study and Multi-Ethnic Study of Atherosclerosis can be requested at https://internal.mesa-nhlbi.org/ and https://chs-nhlbi.org/node/6222, respectively.

## Code availability

The fully trained model and weights are available for download at https://github.com/lst627/CARDIAC-FM.
