# Supplementary material for: CARDIAC-FM: A Multimodal Foundation Model for Cardiovascular Risk Prediction Using ECG and Cardiac MRI
Source: medRxiv. 2026 Mar 18:2026.03.16.26348526. Preprint. [Version 1] doi: 10.64898/2026.03.16.26348526 (PMC13015624; doi:10.64898/2026.03.16.26348526)
Supplement: Supplement 1 [file media-1.pdf]

Statistical Analysis

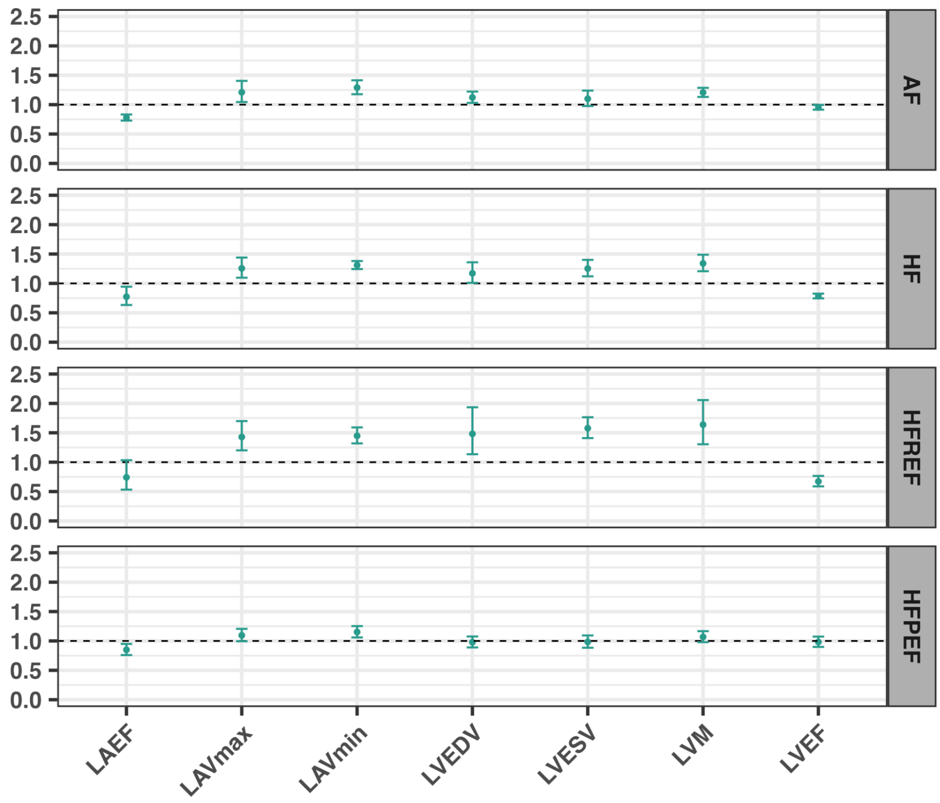

Supplementary Figure 1 | Hazard ratios (95% CI) for zero-shot CARDIAC-FM-predicted CMR features in relation to incident cardiovascular events in the CHS cohort. Predicted CMR parameters include LA ejection fraction (EF), maximum volume, and minimum volume, as well as LV end-diastolic volume (EDV), end-systolic volume (ESV), mass, and EF. Hazard ratios were estimated using Cox proportional hazards models adjusted for traditional cardiovascular risk factors and are expressed per unit increase in each predicted CMR parameter. The dashed line indicates the null value (HR = 1). Error bars represent 95% confidence intervals.

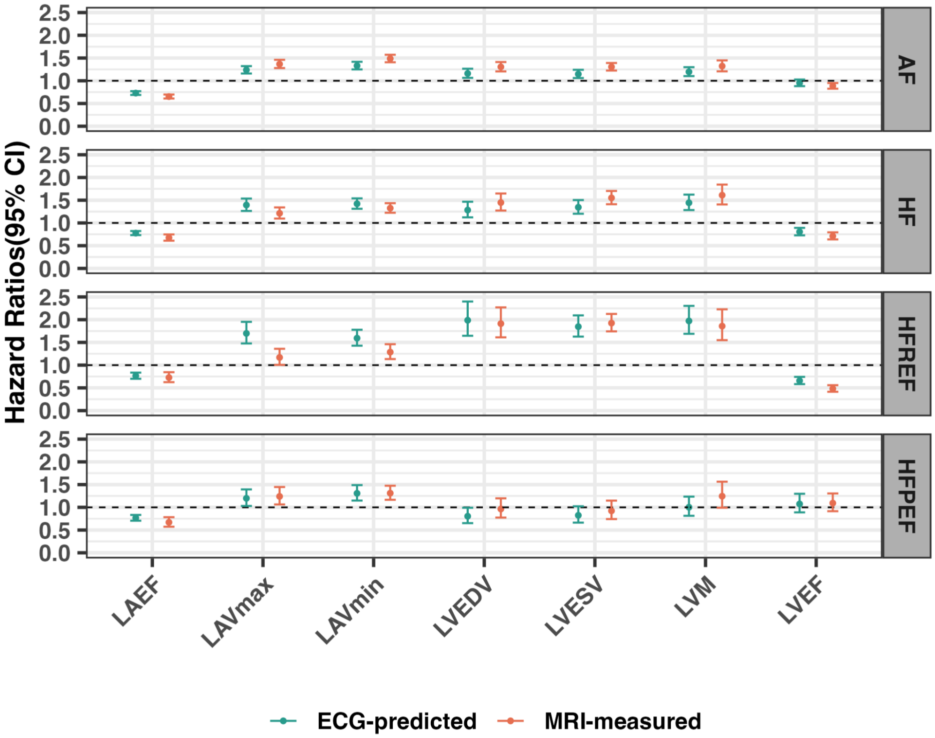

Supplementary Figure 2 | Hazard ratios (95% CI) for zero-shot CARDIAC-FM-predicted and MRI-measured CMR features in relation to incident cardiovascular events in the MESA cohort. CMR parameters include LA ejection fraction (EF), maximum volume, and minimum volume, as well as LV end-diastolic volume (EDV), end-systolic volume (ESV), mass, and EF. Hazard ratios were estimated using Cox proportional hazards models adjusted for traditional cardiovascular risk factors and are expressed per unit increase in each predicted CMR parameter. The dashed line indicates the null value (HR = 1). Error bars represent 95% confidence intervals.

Subgroup Analysis

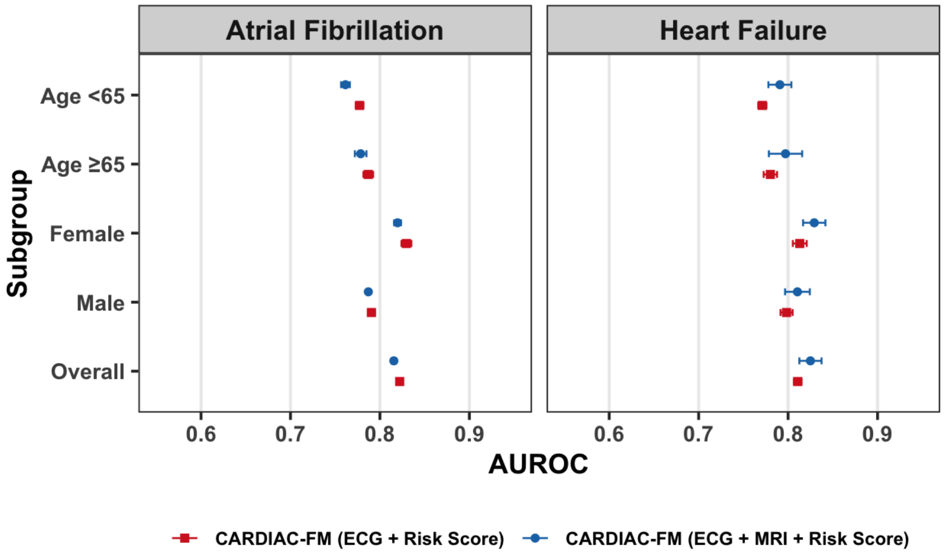

Supplementary Figure 3: Subgroup analysis of CARDIAC-FM performance in the UK Biobank. AUROC for 5-year prediction of incident atrial fibrillation (left) and heart failure (right), stratified by age (<65 versus ≥65 years) and sex. Red points indicate CARDIAC-FM with ECG and clinical risk score; blue points indicate CARDIAC-FM with ECG, MRI, and clinical risk score. Error bars indicate 95% confidence intervals. Both configurations demonstrated consistent discrimination across subgroups.

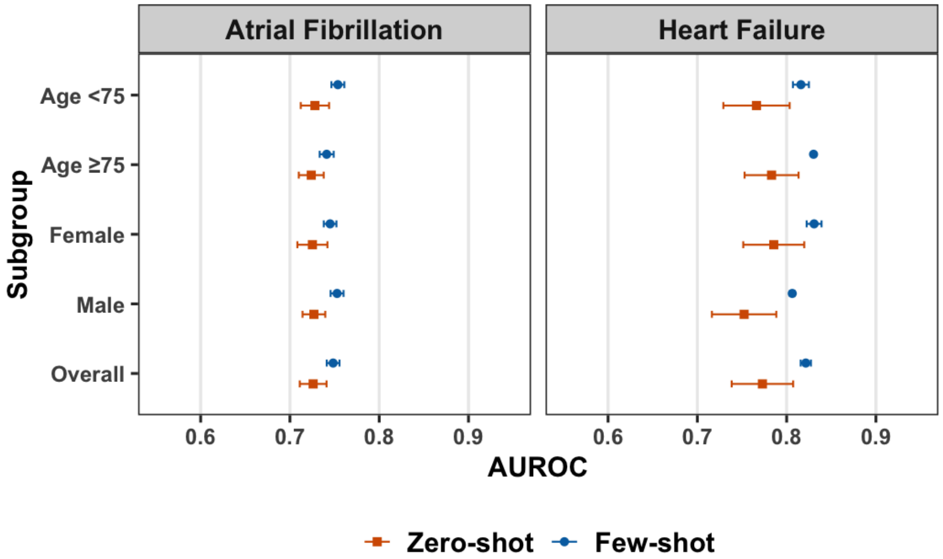

Supplementary Figure 4 | Subgroup prediction for CARDIAC-FM(ECG) in the CHS cohort. AUROC for predicting 3-year and 5-year incident atrial fibrillation (AF) and heart failure (HF). Subgroups include Age (≥75), Age (<75), Female, Male. Error bars indicate 95% confidence intervals.

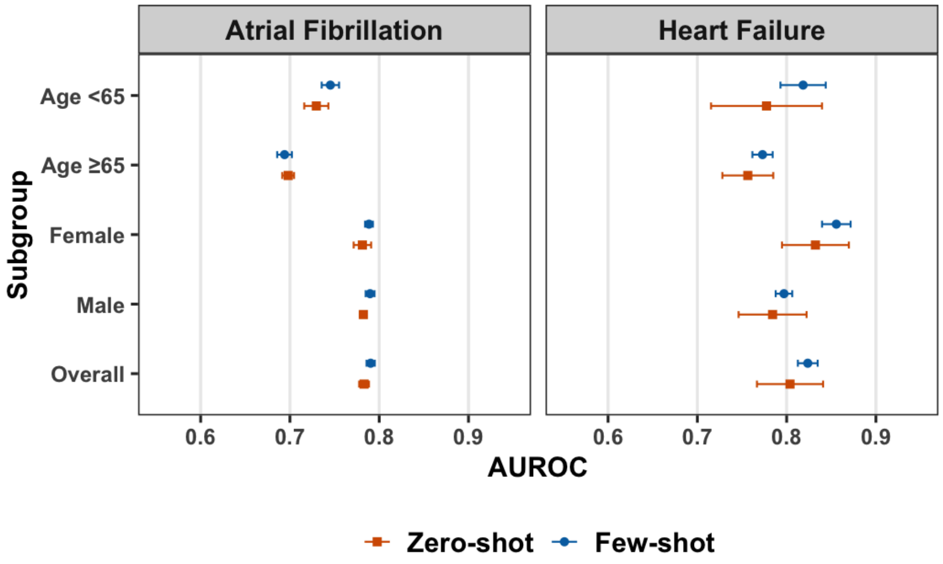

Supplementary Figure 5 | Subgroup prediction for CARDIAC-FM(ECG) in the MESA cohort. AUROC and AUPRC for predicting 5-year incident atrial fibrillation and heart failure. Subgroups include Age (≥65), Age (<65), Female, Male. Error bars indicate 95% confidence intervals.

Cardiac MRI Feature Prediction

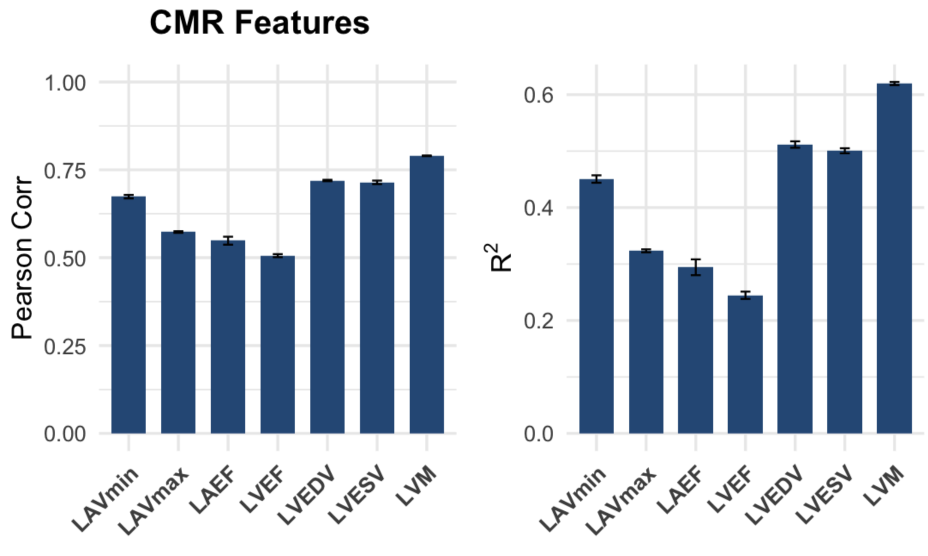

Supplementary Figure 6: Prediction of MRI-derived cardiac phenotypes from ECG using CARDIAC-FM. Pearson correlation coefficients (left) and coefficients of determination (R^2; right) for ECG-based prediction of left atrial minimum volume

(LAVmin), left atrial maximum volume (LAVmax), left atrial emptying fraction (LAEF), left ventricular ejection fraction (LVEF), left ventricular end-diastolic volume (LVEDV), left ventricular end-systolic volume (LVESV), and left ventricular mass (LVM). Error bars represent 95% confidence intervals.
